# Inhibition of SENP5 suppresses cell growth and promotes apoptosis in osteosarcoma cells

**DOI:** 10.3892/etm.2014.1644

**Published:** 2014-03-28

**Authors:** KUN WANG, XIN-CHAO ZHANG

**Affiliations:** Department of Orthopedics, Affiliated Jinshan Hospital, Fudan University, Shanghai 201508, P.R. China

**Keywords:** osteosarcoma, SUMO-specific protease 5, apoptosis, drug target

## Abstract

SUMOylation is a dynamic and reversible process. Several SUMO-specific proteases (SENPs) that remove SUMO from substrates have been shown to be amplified in a subset of cancers. SENP5 is required for cell division, as well as maintaining mitochondrial morphology and function. SENP5 has been reported to be predominantly localized to the cytoplasm of oral squamous cell carcinoma (OSCC) and is associated with the differentiation of OSCC. Western blot was used to detect the protein expression of SENP5 in osteosarcoma cells and tissue. Lentivirus-mediated siRNA was used to silence the expression of SENP5. Cell cycle distribution was determined by FACS analysis. The present study showed that SENP5 is overexpressed in osteosarcoma cells. In addition, lentivirus-mediated small interfering RNA (siRNA) of SENP5 significantly inhibited cell growth and induced apoptosis in osteosarcoma cells. SENP5 inhibition suppressed the growth and colony formation capacity of two osteosarcoma cell lines, U2OS and Saos-2. Silencing the expression of SENP5 in serum-starved U2OS and Saos-2 cells induced an increase in caspase-3/-7 activity and a decrease in cyclin B1 expression. These observations indicate that SENP5 is required for cell growth and apoptosis and may therefore be a promising drug target for antiosteosarcoma treatment.

## Introduction

Osteosarcoma is one of the most common primary bone sarcomas in children and adolescents and has a five-year survival rate of ~70%. Patients with osteosarcoma with metastases at diagnosis have a poor prognosis, with overall survival rates of <20% (1). Despite the rapid development in treatment strategies, the cure rate of patients with osteosarcoma remains extremely poor. Therefore, there is an urgent need to develop novel strategies for the diagnosis and treatment of osteosarcoma.

SUMOylation is an extensively studied modification that elicits a wide range of effects within the cell (2). A small ubiquitin-like modifier (SUMO) protein is covalently attached to lysine residues in substrate proteins in a process similar to ubiquitination (3). SUMO proteins are highly conserved in a large number of species and have been shown to be indispensable in numerous cellular processes. In vertebrates, there are four SUMO family proteins, SUMO-1, -2, -3 and -4. SUMO-2 and SUMO-3 share ~50% identity to SUMO-1, but are highly related to each other, sharing 96% sequence identity (4). SUMO-4 is more similar to SUMO-2/3 (5). SUMOylation is a dynamic and reversible process and six SUMO-specific proteases (SENPs) that remove SUMO from substrates have been identified, which have various substrate specificities and subcellular localizations (6). SENPs are divided into three subfamilies on the basis of sequence homology, cellular location and substrate specificity. The first subfamily consists of SENP1 and SENP2, which have broad substrate specificity. The second subfamily consists of SENP3 and SENP5, which are nucleolar proteins with preferences for SUMO-2/3. The third subfamily consists of SENP6 and SENP7, which have an extra loop in their catalytic domains (7).

DeSUMOylation, induced by SENPs, has been shown to be crucial in determining protein SUMOylation status and activity (8). Several SENPs have been shown to be amplified in a subset of cancer types. Expression of SENP5 has been reported in oral squamous cell carcinoma (OSCC) and the protease has been associated with the differentiation of OSCC (9). SENP5 resides primarily within the nucleoli during interphase, but translocates from the nucleoli to the mitochondrial surface at the G2/M transition, prior to nuclear envelope breakdown (10,11). Knockdown of SENP5 by siRNA results in increased levels of SUMO-1 and SUMO-2/3 conjugates, as well as defects in nuclear and mitochondrial morphology. These observations have revealed an essential role for SENP5 in cytokinesis and the maintenance of mitochondrial function (12–14). However, the biological role of SENP5 in cancer has yet to be fully elucidated.

The present study aimed to determine SENP5 expression levels in osteosarcoma cell lines. In addition, the effect of lentivirus-mediated siRNA of SENP5 on cell growth and apoptosis in osteosarcoma cells was investigated. Furthermore, the study aimed to evaluate whether SENP5, as a SUMO-specific protease, is required for cell growth and apoptosis and may be a promising drug target for antiosteosarcoma treatment.

## Material and methods

### Tissue samples

Primary osteosarcoma and distant normal tissues were collected from routine therapeutic surgeries at the Department of Orthopedics (Affiliated Jinshan Hospital, Shanghai, China). All samples were obtained with informed consent and approved by the Affiliated Jingshan Hospital.

### Cell culture

HOS, KHOS, U2OS, Saos-2 and MG-63 cell lines were purchased from the Cell Bank of Type Culture Collection of the Chinese Academy of Sciences (Shanghai, China) and cultured in RPMI 1640 medium (Gibco-BRL, Beijing, China) supplemented with 10% fetal bovine serum (FBS), 100 IU/ml penicillin and 100 mg/ml streptomycin (Gibco-BRL).

### Quantitative polymerase chain reaction (PCR)

RNA was extracted using TRIzol reagent (Takara Biotechnology Co. Ltd., Dalian, China) and reverse transcription was performed with a Takara RNA PCR kit (Takara Biotechnology Co. Ltd., Dalian, China), in accordance with the manufacturer’s instructions. Quantitative PCR was performed using a SYBR Green Premix *Ex Taq* kit (Takara Bio, Inc., Shiga, Japan), according to the manufacturer’s instructions. PCR was performed in 96-well optical plates. The primers used were as follows: The primers for β-actin 5′-AGAGCTACGAGCTGCCTGAC-3′ and 5′-AGCACTGTGTTGGCGTACAG-3′ and for SENP5 5′-GAGGAAAATTCTATGGAGGA-3′ and 5′-GAGGACAAAGTACTAACATT-3′.

### Western blot analysis

Cells were lysed in sample solution. Proteins were separated on 10% sodium dodecyl sulfate-polyacrylamide gel electrophoresis (SDS-PAGE) gels, transferred to nitrocellulose membranes and detected using various antibodies, as indicated. The membranes were incubated with primary antibodies at 4°C overnight and horseradish peroxidase-conjugated secondary antibodies for 1 h at room temperature, prior to detection using the SuperSignal West Pico Chemiluminescent Substrate kit (Pierce Biotechnology, Inc., Rockford, IL, USA). Anti-β-actin and anti-SENP5 antibodies were purchased from Santa Cruz Biotechnology, Inc. (Santa Cruz, CA, USA) and anti-cyclin B1 antibodies were purchased from Cell Signaling Technology, Inc. (Danvers, MA, USA).

### Cell proliferation assay

U2OS and Saos-2 cells, transfected with mock or SENP5 siRNA, were seeded in 96-well plates and incubated for one to six days. Subsequently, 20 μl 3-(4,5-dimethylthiazol-2-yl)-2, 5-diphenyltetrazolium bromide solution (5 mg/ml) was added to each well 3 h prior to the end of incubation. The crystals were dissolved in 150 μl dimethyl sulfoxide and the absorbance at 570 nm was measured with a SPECTRAmax 340PC (Molecular Devices, LLC., Sunnyvale, CA, USA).

### Colony formation assay

U2OS and Saos-2 cells, transfected with mock or SENP5 siRNA, were seeded in a six-well plate at a density of 500 or 1,000 cells/well. Following incubation at 37°C for 12–21 days, the colonies were fixed and stained in a dye solution containing 0.1% crystal violet (Sigma-Aldrich, St. Louis, MO, USA) and 20% methanol. The number of colonies per well was counted.

### Cell cycle analysis

Cells grown in regular growth medium for 24 h were collected, fixed in 70% cold ethanol overnight and stained with phosphate-buffered saline (PBS) containing 50 μg/ml propidium iodide and 100 μg/ml RNase A for 30 min at 37°C. The DNA content of the labeled cells was measured using the Accuri C6 flow cytometry system (BD Biosciences, Franklin Lakes, NJ, USA).

### Analysis of caspase-3/-7 activity

U2OS and Saos-2 cells, transfected with mock or SENP5 siRNA, were seeded at a density of 500 cells/well in triplicate wells in a 384-well plate. Following overnight incubation, the medium was replaced with Dulbecco’s modified Eagle’s medium (DMEM) supplemented with 0.2% FBS and incubated for an additional 48 h. Caspase activity was subsequently measured with a Caspase-Glo 3/7 Assay System (Promega, Fitchburg, WI, USA), according to the manufacturer’s instructions. An equal volume of caspase substrate was added to the cells and the samples were incubated at room temperature for 1 h. Luminescence was measured using an EnVision 2103 Multilabel Reader (Perkin Elmer, Inc., Waltham, MA, USA). Luminescence of the mock-transfected cells was set as the standard.

### Statistical analysis

The data shown represent the mean ± standard error (SE) values of three independent experiments. Significance was analyzed using a Student’s t-test. P<0.05 was considered to indicate a statistically significant result.

## Results

### SENP5 is overexpressed in osteosarcoma cell lines and tissues

**Figure 1:**
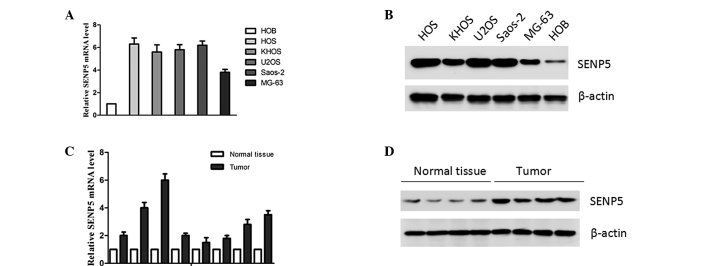
SENP5 is overexpressed in osteosarcoma cell lines (HOS, KHOS, U2OS, Saos-2 and MG-63) and tissues. (A) mRNA expression and (B) protein levels of SENP5 in indicated osteosarcoma cell lines. (C) mRNA expression of SENP5 in eight paired clinical specimens and (D) protein levels of SENP5 in four paired clinical specimens. SENP5, SUMO-specific protease 5; HOB, human osteoblasts isolated from normal human bone.

To investigate the significance of SENP5 in osteosarcoma carcinogenesis, the expression levels of SENP5 in osteosarcoma cell lines (HOS, KHOS, U2OS, Saos-2 and MG-63) and clinical specimens were analyzed using quantitative PCR and western blotting. The results showed that SENP5 was significantly overexpressed in all osteosarcoma cell lines, compared with HOB cells (human osteoblasts isolated from normal human bone) (Fig. 1A and B). Consistent with these observations, osteosarcoma clinical specimens expressed high levels of SENP5 compared with adjacent normal bone tissues (Fig. 1C and D). These results indicated that increased expression of SENP5 was correlated with osteosarcoma carcinogenesis.

### Lentivirus-mediated siRNA of SENP5 significantly inhibits cell growth in osteosarcoma cells

**Figure 2:**
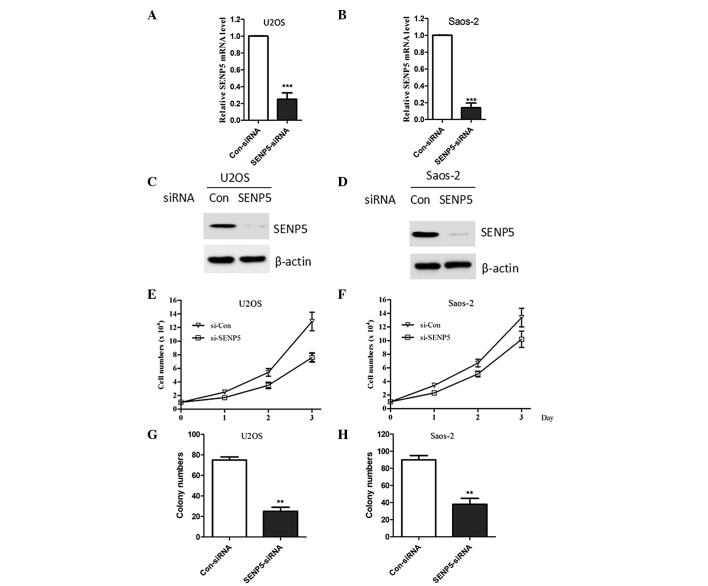
Lentivirus-mediated siRNA of SENP5 significantly inhibits cell growth in osteosarcoma cells. mRNA levels of SENP5 in (A) U2OS and (B) Saos-2 cells transfected with mock or SENP5 siRNA. Protein levels of SENP5 in (C) U2OS and (D) Saos-2 cells transfected with mock or SENP5 siRNA. Growth curves of (E) U2OS and (F) Saos-2 cells transfected with mock or SENP5 siRNA. Relative colony counts for (G) U2OS and (H) Saos-2 cells transfected with mock or SENP5 siRNA. The amount of mock siRNA cell colonies was set as 100%. SENP5, SUMO-specific protease 5; siRNA, small interfering RNA; Con, control (mock). ^*^P<0.05, ^**^P<0.01, ^***^P<0.001.

To investigate the biological role of SENP5 in osteosarcoma, lentivirus-mediated siRNA was utilized to silence the expression of endogenous SENP5 in osteosarcoma cells. To testify the silencing effect of lentivirus-mediated siRNA targeting the SENP5 gene, quantitative PCR and western blot analysis were performed to detect the expression of SENP5 mRNA and protein in mock or stably transfected U2OS and Saos-2 cells. As shown in Fig. 2A–D, mRNA and protein levels of SENP5 significantly decreased in SENP5-silenced U2OS and Saos-2 cells. These results indicated that the lentivirus-mediated RNAi system was able to effectively knockdown endogenous SENP5 expression in osteosarcoma cells. Silencing the expression of SENP5 significantly decreased the proliferation of U2OS and Saos-2 cells (Fig. 2E and F). Consistent with these observations, silencing the expression of SENP5 resulted in a marked decrease in the number and size of U2OS and Saos-2 cell colonies (Fig. 2G and H).

### SENP5 inhibition results in G2/M arrest and apoptosis in U2OS and Saos-2 osteosarcoma cells

**Figure 3:**
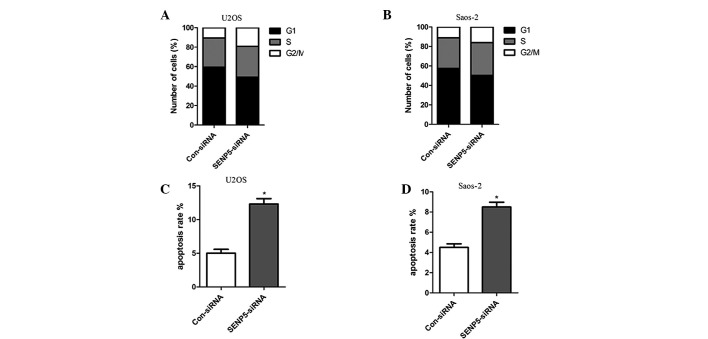
SENP5 inhibition results in G2/M arrest and apoptosis in U2OS and Saos-2 osteosarcoma cells. Cell cycle distribution of (A) U2OS and (B) Saos-2 cells transfected with mock or SENP5 siRNA. Cells were fixed with 70% ethanol and stained with PI prior to FACS analysis. (C) U2OS and (D) Saos-2 cells, transfected with mock or SENP5 siRNA, were stained with annexin V/PI and apoptotic cells were determined by FACS analysis. SENP5, SUMO-specific protease 5; siRNA, small interfering RNA; PI, propidium iodide; FACS, fluorescence-activated cell sorting; Con, control (mock). ^*^P<0.05.

The growth inhibition of cells may be caused by a reduced cell proliferation rate or by increased apoptosis or cell cycle arrest. The effect of SENP5 on cell cycle distribution was investigated. Silencing the expression of SENP5 caused a significant increase in the number of U2OS and Saos-2 cells in the G2/M phase (Fig. 3A and B), which was consistent with the previously identified role of SENP5 in cell division. Moreover, SENP5 inhibition also resulted in spontaneous osteosarcoma cell apoptosis, when compared with mock inhibition cells (Fig. 3C and D). In combination, these results indicated that SENP5 inhibition decreased osteosarcoma cell proliferation by inducing G2/M arrest and apoptosis.

### SENP5 inhibition induces caspase-3/-7 activity and inhibits cyclin B1 expression

**Figure 4:**
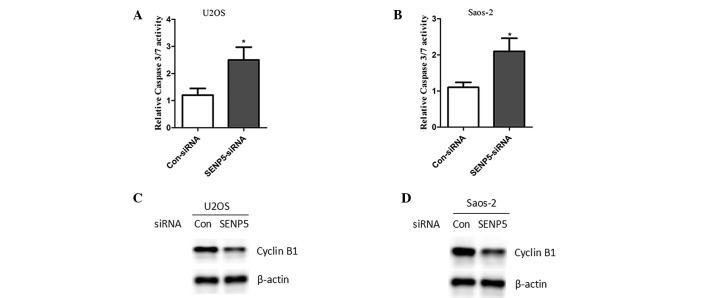
SENP5 inhibition induces caspase-3/-7 activity and inhibits cyclin B1 expression. Caspase-3/-7 activity of (A) U2OS and (B) Saos-2 cells transfected with mock or SENP5 siRNA. Cells were plated at a density of 500 cells/well in a 384-well plate and incubated in medium containing 0.2% FBS for two days, prior to analysis with Caspase-Glo 3/7 assay kits. Protein levels of cyclin B1 in (C) U2OS and (D) Saos-2 cells transfected with mock or SENP5 siRNA. SENP5, SUMO-specific protease 5; siRNA, small interfering RNA; FBS, fetal bovine serum; Con, control (mock). ^*^P<0.05.

To investigate the mechanism of SENP5 in the regulation of osteosarcoma cell apoptosis, it was assessed whether SENP5 inhibition resulted in the activation of caspases. The caspase-3/-7 activity of mock- or SENP5-depleted osteosarcoma cells was evaluated. These cells were serum-starved in 0.2% FBS for two days. The caspase-3/-7 activity in mock-depleted cells was set as the standard. SENP5 inhibition resulted in a two-to-three-fold increase in caspase-3/-7 activity in U2OS and Saos-2 cells (Fig. 4A and B). The mechanism of SENP5-depletion-induced G2/M arrest in osteosarcoma cells was also investigated. It was observed that silencing the expression of SENP5 significantly decreased the expression of cyclin B1, a key regulator of G2/M transition in the cell cycle (Fig. 4B and C).

## Discussion

SUMOylation has a vital role in tumors (15), with several SENPs identified to be involved in cancer development. SENP1 has been shown to be crucial in the development of prostate cancer by modulating the SUMOylation of the androgen receptor (16). SENP2 has been shown to be involved in hepatocellular carcinoma cell growth by modulating the stability of β-catenin (17). SENP3 has been identified to accumulate in a variety of types of primary human cancer, including colon adenocarcinoma, by modulating the SUMOylation status of the tumor suppressor, promyelocytic leukemia protein (PML) (18). SENP6 was previously reported to induce radiosensitization of hepatocellular carcinoma cells by blocking radiation-induced NF-κB activation (19).

In the present study, SENP5 was observed to be overexpressed in osteosarcoma cell lines and tissues. SENP5 primarily resides within the nucleoli during interphase, but translocates from the nucleoli to the mitochondrial surface at the G2/M transition, prior to nuclear envelope breakdown (14). Function studies have revealed that SENP5 is required for cell division and the maintenance of mitochondrial morphology and function (11,14). In the present study in osteosarcoma cell lines, it was observed that silencing the expression of SENP5 significantly decreased cell proliferation, which was consistent with the function of SENP5 in cell division. The results indicated that SENP5 regulated osteosarcoma cell proliferation by inducing G2/M arrest and apoptosis. Further elucidation of the underlying mechanisms was also achieved, and it was demonstrated that SENP5 inhibition increased the activity of caspase-3/-7 and decreased the expression of cyclin B1. Thus, the present study reported a role of SENP5 in the regulation of osteosarcoma cell proliferation and apoptosis.

Although the deSUMOylation activity of SENP5 has been well-documented, the endogenous SUMOylation substrates of SENP5 are less well-known. The first substrate of SENP5 identified was the tumor suppressor, PML, which has an essential role in the regulation of cell proliferation (10). DRP1, a mitochondrial fission GTPase, was then identified to be a substrate of SENP5, and SENP5 translocates from the nucleoli to the mitochondria and modulates DRP1-dependent fission during mitosis (11). However, substrates in osteosarcoma cells that are responsible for SENP5-regulated G2/M arrest and apoptosis remain to be identified. The results from the present study reveal an indispensable role of SENP5 in the regulation of G2/M arrest and apoptosis in osteosarcoma cells, which supports the hypothesis that SENP5 may be a promising drug target for antiosteosarcoma treatment in the future.
